# A Pragmatic Approach to Assessment of Chronic and Recurrent Pain in Children with Severe Neurologic Impairment

**DOI:** 10.3390/children9010045

**Published:** 2022-01-02

**Authors:** Simone Benvenuto, Andrea Trombetta, Egidio Barbi

**Affiliations:** 1 Department of Medical, Surgical and Health Sciences, University of Trieste, 34127 Trieste, Italy; simone.benvenuto2@icloud.com; 2 IRCCS Materno Infantile Burlo Garofolo, University of Trieste, 34137 Trieste, Italy; egidio.barbi@burlo.trieste.it

**Keywords:** pain, severe neurologic impairment

## Abstract

The term “severe neurologic impairment” (SNI) is used to describe a group of disorders of the central nervous system which arise in childhood, resulting in motor impairment, cognitive impairment and medical complexity. As a result, much assistance is required with activities of daily living. Since these patients are often unable to self-report pain, or they may exhibit uncommon behaviors when suffering, pain manifestations may go unrecognized. In this article, the basic principles of how to approach pain in children with SNI are discussed.

## 1. Introduction

Severe neurologic impairment (SNI) describes a group of disorders of the central nervous system that arise in childhood, resulting in motor and cognitive impairment, along with medical complexity, where much assistance is required with activities of daily living. The impairment is permanent and can be progressive or static [1]. Several conditions may cause SNI, such as genetic syndromes, traumatic brain injury, neurodegenerative disorders, epileptic syndromes and cerebral palsy, with the latter being the most frequent with an incidence of two patients for each 1000 of pediatric age [2]. It is well-known that these patients experience pain more frequently than healthy peers, mostly not due to accidental injury [3], but rather to their condition’s related morbidities and iatrogenic pain [4]. Indeed, iatrogenic pain (being the cause of up to 10% of pain in this population [5]) that is caused by painful procedures that are often repeated, such as venepuncture, botulinum toxin injections or surgery, should be carefully managed.

While being a frequent problem and being outlined by several pain behaviors and autonomic responses, such as restlessness, tachycardia and sharp breathing, pain may go unrecognized, since these patients are often unable to self-report it or may exhibit uncommon behaviors when suffering [6]. Furthermore, there is evidence that the brain cortex in children with SNI has higher sensitivity to external stimuli, due to altered somatosensory brain processing, with a lower pain threshold [7]. Physicians should be fully aware of these issues, since untreated chronic or recurrent pain profoundly affects the quality of life of these children by affecting sleep [8], mood and their social skills, as well as their physical and cognitive abilities [9]. Moreover, untreated pain interferes with their routine activities and adaptive function and can significantly worsen the major functional limitations associated with their neurological condition. Finally, children’s undiagnosed and untreated pain may increase parents and caregivers’ worries, frustration and anxiety, thus worsening an already challenging quality of life [10]. The basic principles of how to approach pain in children with SNI are discussed in a pragmatic and concise way.

## 2. Pain Recognition and Measurement

Specific pain assessment scales have been introduced, based on the observation of pain behaviors, such as the Non-Communicating Children’s Pain Checklist-Postoperative Version (NCCPC-PV) and the NCCPC-Revised (NCCPC-R); the Individualized Numeric Rating Scale (INRS); the Pediatric Pain Profile (PPP); and the revised Face, Legs, Activity, Cry, Consolability (R-FLACC) scale. The main advantage of these scales is that they include physiologic (tachycardia, shivering and breath-holding) and behavioral items (clenching or grinding teeth; flinching or moving the body part away) in order to aim at complete pain recognition. The main disadvantage is that they require some specific training and are time-consuming. There is no evidence in the literature about which scale performs better; however, all are considered to be more precise than generic pain-assessment tools. The NCCPC-PV is based on 27 items, with a sensitivity of 0.88 and a specificity of 0.81 in identifying moderate-to-severe pain, using a cutoff of ≥11 out of 81 points [11]. In its revised version (NCCPC-R), based on 30 items, a cutoff of ≥7 of 90 provided a sensitivity of 0.84 and a specificity of 0.77 [12]. Both of these scales are standardized tools, with the main advantage of having an excellent inter-rater reliability. The INRS is a personalized tool, with a score ranging from 0 to 10, showing good reliability (0.82–0.87) when simultaneously used by parents and nurses, and a moderate validity (0.63–0.73) when compared to NCCPC-PV [13]. The PPP is based on 20 items, showing its best sensitivity (1.00) and specificity (0.91) at a cutoff of 14/60, and an inter-rater reliability of 0.74–0.89 [14]. Finally, the R-FLACC scale considers five items (Table 1), with a maximum score of 10, and an inter-rater reliability of 0.76–0.90 [15]. It requires a previous agreement and identification with the caregiver of each child-specific pain behavior. In a study, nurses and physicians rated this scale as having higher clinical utility in terms of complexity, compatibility and relative advantage when compared to the Non-Communicating Children Pain Checklist-Postoperative Version (NCCPC-PV) [16]. The latter may be considered in the absence of a predefined pain assessment with parents, as required by the R-FLACC. While the use of these scales allows individualization of each child-specific pain behaviors, such as the misleading laughter, “freezing” or self-injurious behaviors, they may also be a precious tool to establish a shared knowledge and common language with parents. A more extensive use of these scales may help go beyond false beliefs, such as the alleged indifference to the pain of some of these children. 

We are not aware of studies comparing the use of specific pain scale versus parents’ opinion. However, pain assessment and management in these children should always include a positive interaction with their parents, who remain the best proxy measure of their kids’ pain [17]. A milestone paper [18] described how experiential learning can lead mothers to “develop a sense of knowing” their children, becoming competent interpreters and translators of their children’s pain. Health professionals, through an empathic attitude and ability to listen, should support this process so that recognition and action based on parent’s concerns will help reach the best pain-related decisions about their children.

**Figure 1:**
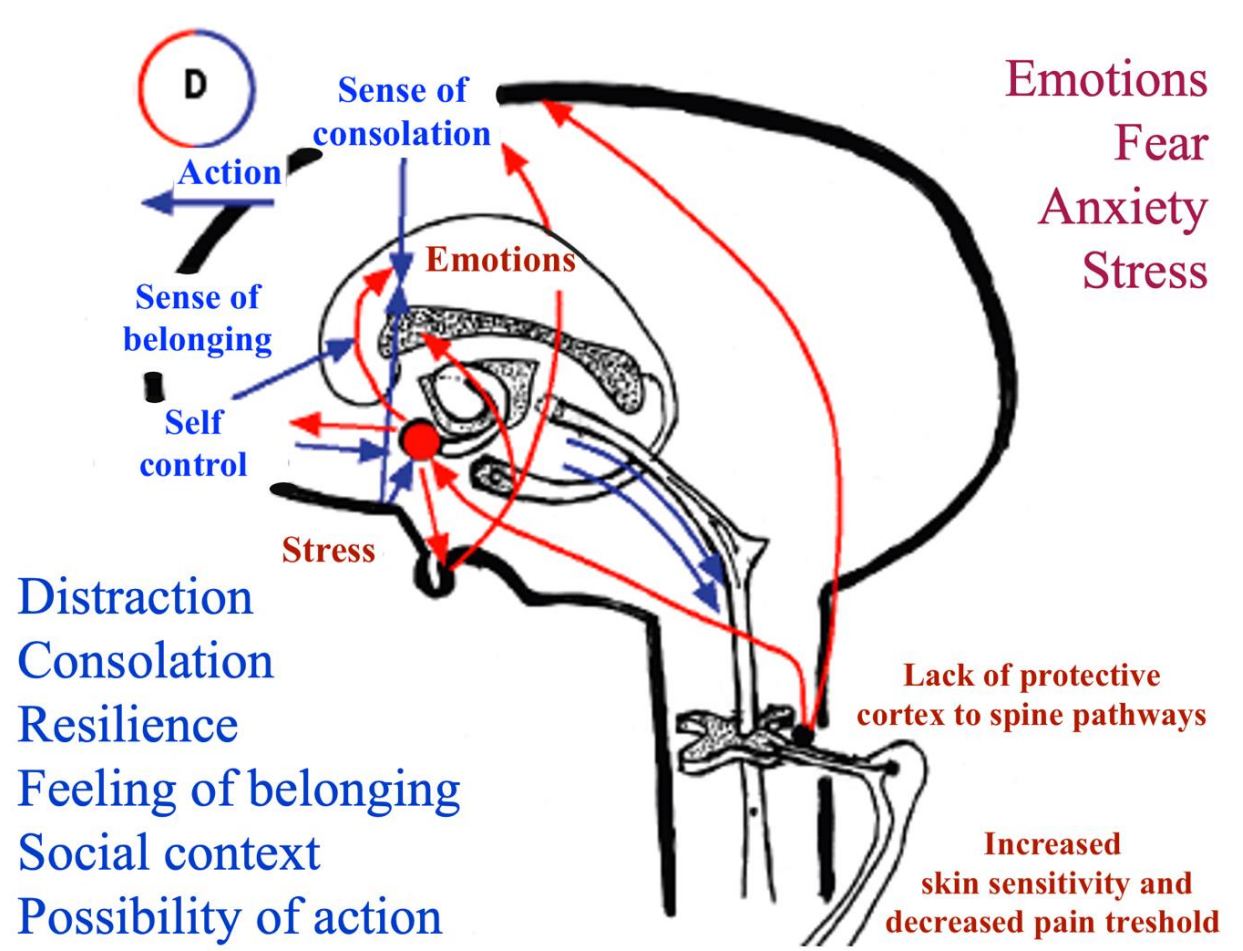
Pain and antinociceptive system pathways. Pain amplification (red) and control (blue) factors. Modified from Panizon and Barbi. Some Specific Issues on Pain in Pediatrics: Summary and Review of the Present Knowledge and Practice. Medico e Bambino, 2010; 29:289–297. See also Reference [7].

The role of parents is also crucial in distinguishing fear and anxiety from pain. In children with moderate-to-severe cognitive impairment, anxiety and fear have been shown to play an essential role in procedural pain and worsen its perception and impact. Especially in the setting of procedural pain, the relevance of fear and anxiety should not be underestimated and should be approached by mean of non-pharmacologic techniques and parents’ active involvement [19]. Notably, parents’ behavior impacts the quality of the child’s procedural experience and pain. Therefore, decreasing parental anxiety may also contribute to the reduction of the child’s anxiety, and vice versa [20]. A recent study demonstrated a significantly different cortical activation pattern during venipuncture in children with SNI, as compared to healthy peers. This evidence suggests that the need for physical restraint and a possible lack of frontal to limbic areas’ connection may cause an impaired control of emotions with a worsening of pain perception [21]. Indeed, cortical damage in these children may impair cortical antinociceptive signals acting both on limbic structures and on spinal pain transmission gates through descending protective pathways. The possible factors playing a role in the perception of pain in children with SNI are summarized in Figure 1.

## 3. Diagnostic Workup for Pain in Patients with Cognitive Impairment

**Figure 2:**
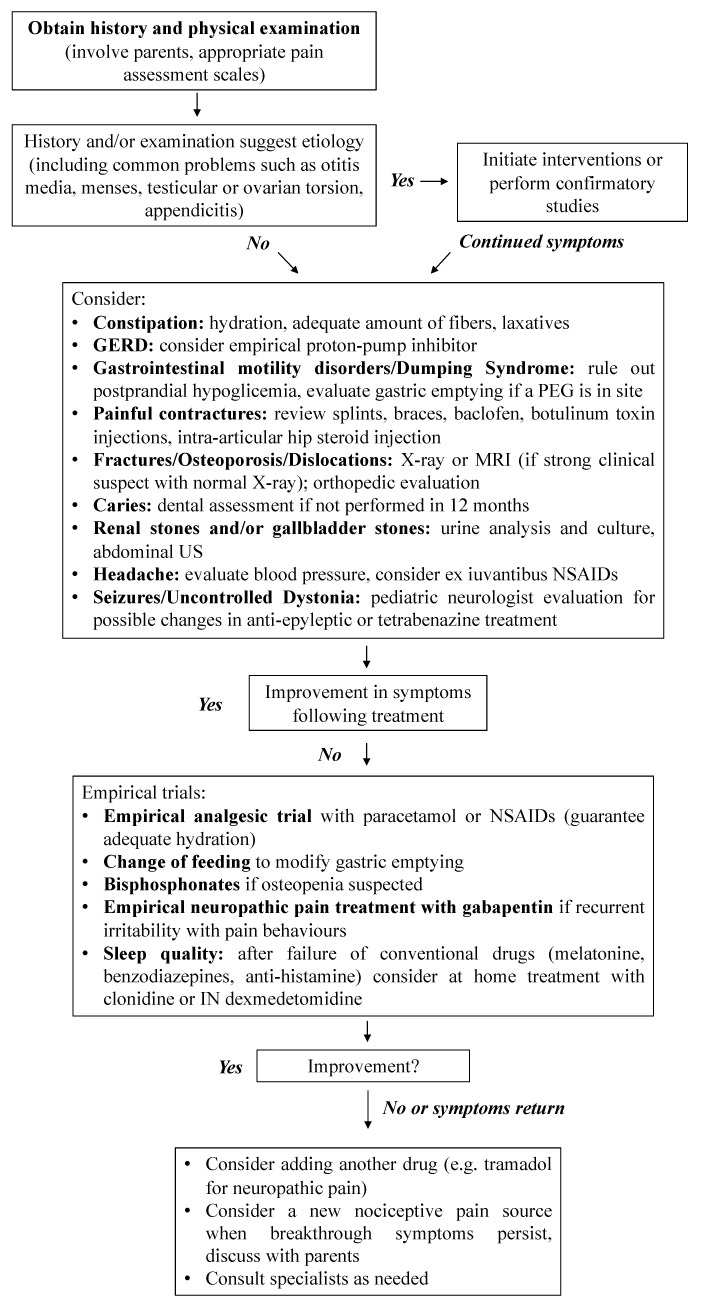
Diagnostic workup for pain in patients with cognitive impairment. Modified from Hauer and, Houtrow, Pain Assessment and Treatment in Children with Significant Impairment of the Central Nervous System. Pediatrics 139 (2017).

The most frequent possible causes of pain in this subset of patients have already been well described in the literature [4]. Therefore, a thorough re-definition of each cause goes beyond the scope of the paper. In a pragmatic and basic approach, we suggest a possible diagnostic workup for pain in these patients, based on a problem-oriented approach (Figure 2). We also discuss first-line intervention to manage this issue, along with major pitfalls pediatricians may encounter in their routine daily practice and when assessing children with SNI.

### 3.1. Gastrointestinal Tract

The gastrointestinal tract is one of the most common sources of pain among these patients. Impaired gastrointestinal motility, insufficient hydration and immobility lead to constipation in up to 75% of patients [22]. An adequate amount of fibers should be provided to them, but there is a very fine balance, because high-fiber formulas can slow down gastric emptying.

As a matter of fact, gastric emptying in children with a percutaneous endoscopic gastrostomy (PEG) and Nissen fundoplication may be delayed, thus causing pain and nausea. On the one hand, an extensively hydrolyzed formula has been shown to accelerate gastric emptying and limit gastro-esophageal reflux in children previously fed with a 100% casein formula [23]. On the other hand, an amino acidic feeding may cause an unpleasant sense of gastric emptying or even facilitate a dumping syndrome, which may represent a common complication in this category of children [24]. Even if it is empirically recommended, no evidence is available in the literature about the effectiveness of blended food in this context. While not being painful, a dumping syndrome could evoke either pain or an epilepsy in a non-verbal child. Indeed, this should be considered in any irritable, sweaty and restless child with a PEG two hours after the meal and confirmed by detection of hypoglycemia. In the perspective of a gastric motility disorder, a trial with a different feeding should be considered [25].

Gastro-esophageal reflux disease should be considered an unlikely cause of pain in patients already treated with adequate dosage proton-pump inhibitors, whose therapy can be continued indefinitely [26]. An amino acidic formula may improve reflux in selected patients with no benefit from a previous surgical option, such as a fundoplication procedure or a jejunal tube. If present, the PEG tube should be examined to rule out gastrostomy infections, granuloma, dislocation, occlusion or even a buried bumper syndrome in cases of obstruction.

Iatrogenic intervention on bowel motility (i.e., nasogastric tube, jejunal tube or PEG insertion) may also induce repeated mechanical stimuli in a dysmotile gastrointestinal tract [27]. Nociceptive signals from sensitized spinal afferents can lead to a progressive buildup in cumulative depolarization, known as the “wind-up phenomenon”, resulting in central sensitization and therefore contributing to restlessness, sleeplessness and nausea [28].

### 3.2. Musculoskeletal Pain

The issue of hypertonia, including spasticity, dystonia and non-specific back pain, should be managed with a child neurologist and physiotherapist in order to optimize the use of splints and braces and to evaluate the need for anti-dystonic medications or botulinum toxin treatment.

Intractable pain due to a dislocated hip may be approached with an intra-articular steroid and a topical anesthetic injection, which may allow months of well-being [29].

Osteopenia is found in up to 95% of non-ambulating children with cognitive impairment, and up to 20% will experience a femoral fracture during their life [30], with a definite risk of recurrence. Radiography should be performed to rule out fractures or hip dislocations when positioning, bathing or dressing is difficult because of the pain, while MRI should be considered in cases of strong clinical suspect with normal X-ray to rule out occult fractures or osteomyelitis. The use of bisphosphonates is formally restricted after the occurrence of one or two fractures. If osteopenia-related back pain is suspected, especially in the case of pain that worsens at night or while the child is moved, a bone densitometry and an ex juvantibus trial with bisphosphonates should be considered [31]. However, clinicians should be aware that the use of these drugs in children with SNI remains controversial [32] and adverse effects such as gastrointestinal reflux, “flu-like” symptoms, hypocalcemia and delay in bone healing after an orthopedic procedure such as osteotomy have been reported [33].

### 3.3. Other Common Source of Pain

A dental assessment should be deemed if not already performed in the past year, even when no specific concerns are identified. Urinalysis and culture to detect an infection and abdominal US to rule out renal and/or gallbladder stones should be systematically performed.

### 3.4. Neurogenic and Other Central Nervous System Causes of Pain

A child neurologist with specific experience in the field should always be consulted when a cause for pain is not identified; to rule out and treat possible dystonic disorders; or when rarer epileptic equivalents that may need a specific therapeutic approach, such as tetrabenazine [34].

If the above causes are ruled out, an empirical medication trial directed to neuropathic pain could be considered in children with long-standing irritability and pain behaviors. Remarkably, some genetic disorders, such as the Noonan syndrome, are specifically characterized by a higher risk of neuropathic pain [35]. On the contrary, children with Prader Willi syndrome may typically display a higher pain threshold. Gabapentin is safe and effective on peripheral and central neuropathic pain, autonomic dysfunction, visceral hyperalgesia and spasticity in adults, so it represents a reasonable first-line choice; however, it is off-label for children [10]. The benefit of such a therapy may also indirectly confirm the neuropathic etiology of pain.

Children with chronic or recurrent pain may suffer from impaired sleep quality [36]. Notably, a sleep disorder can also be mistaken for chronic pain, due to their reciprocal influence [37], and a trial with melatonin could be started. In cases of severe pain and dystonia, with no benefit from any conventional pharmacological treatment, clonidine administered through an epidural, intrathecal and local/topical route may be effective in chronic pain conditions where neuropathy is a predominant component. In the setting of future trends of research, the symptomatic intranasal use of dexmedetomidine at home has been anecdotally reported in the context of palliative care [38,39]. In the setting of chronic pain (persistent, with recurrence of more than 3 months), some authors suggest that no routine diagnostic tests should be carried out, and the goal should be solely directed to the pain relief [40]. While this concept fits well with children who had a reasonable diagnostic workout, it should also be considered that a diagnosis of a specific cause may be of great relevance, not only to allow a specific treatment (for example, an antibiotic treatment for struvite renal stones) but also for parents. As a matter of fact, a diagnosis and the comprehension of what is happening to the child may limit parents’ anxiety and fear for an undefined condition, and help them to seek support.

### 3.5. Do Not Forget: Non-Pharmacological Interventions

Finally, as a general rule for the management of pain in pediatric-age patients, and especially in children with SNI, clinicians should always consider the role of non-pharmacological interventions for pain relief, with strategies such as cuddling, tight swaddling, repositioning, warm baths and massage [41]. Adequate treatment of pain in these children may become a complex process, requiring a lot of time and several professional figures to adequately identify and address the often-overlapping sources of pain. In some cases, little improvement is obtained despite multiple trials, and the balance between potential benefits and the risk of over-testing needs to be discussed with parents. A prompt recognition and treatment of pain in children with SNI could therefore not only improve their daily quality of life but possibly prevent chronicization of pain until it becomes intractable.

## Figures and Tables

**Table 1:** Revised Face, Legs, Activity, Cry, Consolability (R-FLACC) scale (from Malviya et al. The revised FLACC observational pain tool: improved reliability and validity for pain assessment in children with cognitive impairment. Paediatr Anaesth. 2006; 16(3): 258–265).

**Face**
0 = No particular expression or smile 1 = Occasional grimace/frown; withdrawn or disinterested; appears sad or worried 2 = Consistent grimace or frown; frequent/constant quivering chin, clenched jaw; distressed-looking face; expression of fright or panic Individualized behavior:___________
**Legs**
0 = Normal position or relaxed; usual tone and motion to limbs 1 = Uneasy, restless, tense; occasional tremors 2 = Kicking, or legs drawn up; marked increase in spasticity, constant tremors or jerking Individualized behavior:___________
**Activity**
0 = Lying quietly, normal position, moves easily; regular, rhythmic respirations 1 = Squirming, shifting back and forth, tense or guarded movements; mildly agitated (e.g., head back and forth and aggression); shallow, splinting respirations, intermittent sighs 2 = Arched, rigid or jerking; severe agitation; head banging; shivering (not rigors); breath holding, gasping or sharp intake of breaths, severe splinting Individualized behavior:___________
**Cry**
0 = No cry/verbalization 1 = Moans or whimpers; occasional complaint; occasional verbal outburst or grunt 2 = Crying steadily, screams or sobs, frequent complaints; repeated outbursts, constant grunting Individualized behavior:___________
**Consolability**
0 = Content and relaxed 1 = Reassured by occasional touching, hugging or being talked to. Distractible 2 = Difficult to console or comfort; pushing away caregiver, resisting care or comfort measures Individualized behavior:___________

## Abbreviations

GERD: Gastroesophageal Reflux Disease. INRS: Individualized Numeric Rating Scale. MRI: Magnetic Resonance Imaging. NCCPC-R: Non-Communicating Children Pain Checklist-Revised. NSAIDs: Nonsteroidal Anti-Inflammatory Drugs. PPP: Paediatric Pain Profile. PEG: Percutaneous Endoscopic Gastrostomy. R-FLACC: revised Face, Legs, Activity, Cry, Consolability. SNI: Severe Neurologic Impairment. US: Ultrasound Scan.
